# Supplementary material for: Peta-bit-per-second optical communications system using a standard cladding diameter 15-mode fiber
Source: Nat Commun. 2021 Jul 9;12:4238. doi: 10.1038/s41467-021-24409-w (PMC8270968; doi:10.1038/s41467-021-24409-w)
Supplement: Supplementary file 2 — Author contacts [file 41467_2021_24409_MOESM2_ESM.pdf]

Georg Rademacher, corresponding author

georg.rademacher@nict.go.jp

Benjamin J. Puttnam

ben@nict.go.jp

Ruben S. Luís

rluis@nict.go.jp

Tobias A. Eriksson

teriksson@infinera.com

Now with Infinera, Sweden

Yoshinari Awaji

yossy@nict.go.jp

Hideaki Furukawa

furukawa@nict.go.jp

National Institute of Information and Communications Technology, Tokyo, Japan

Nicolas K. Fontaine,

nicolas.fontaine@nokia-bell-labs.com

Mikael Mazur

mikael.mazur@nokia-bell-labs.com

Haoshuo Chen,

haoshuo.chen@nokia-bell-labs.com

Roland Ryf,

roland.ryf@nokia-bell-labs.com

David T. Neilson

David.neilson@nokia-bell-labs.com

Nokia Bell Labs, New Jersey, USA

Pierre Sillard

Pierre.sillard@prysmiangroup.com

Prysmian, France

Frank Achten

Frank.achten@prysmiangroup.com

Prysmian, Netherlands
